# Evaluation of 3-(3-chloro-phenyl)-5-(4-pyridyl)-4,5-dihydroisoxazole as a Novel Anti-Inflammatory Drug Candidate

**DOI:** 10.1371/journal.pone.0039104

**Published:** 2012-06-18

**Authors:** Amanda Roberta Revoredo Vicentino, Vitor Coutinho Carneiro, Anderson de Mendonça Amarante, Claudia Farias Benjamim, Alcino Palermo de Aguiar, Marcelo Rosado Fantappié

**Affiliations:** 1 Instituto de Bioquímica Médica, Programa de Biologia Molecular e Biotecnologia, Universidade Federal do Rio de Janeiro, CCS, Ilha do Fundão, Rio de Janeiro, Brasil; 2 Instituto de Ciências Biomédicas, Departamento de Farmacologia, Universidade Federal do Rio de Janeiro, CCS, Ilha do Fundão, Rio de Janeiro, Brasil; 3 Instituto Militar de Engenharia, Departamento de Química, Praça General Tibúrcio, 80, Rio de Janeiro, Brasil; Universidad Federal de Santa Catarina, Brazil

## Abstract

**Background:**

3-(3-chloro-phenyl)-5-(4-pyridyl)-4,5-dihydroisoxazole (DIC) is a five-membered heterocyclic compound containing a N-O bond. The anti-inflammatory effects of this compound were studied both *in vitro* and *in vivo*.

**Principal Findings:**

DIC effectively decreased TNF-α and IL-6 release from LPS-stimulated macrophages in a dose dependent manner. DIC diminished the levels of COX-2 with subsequent inhibition of PGE_2_ production. DIC also compromised HMGB1 translocation from the nucleus to the cytoplasm. Moreover, DIC prevented the nuclear translocation of NF-κB and inhibited the MAPK pathway. *In vivo*, DIC inhibited migration of neutrophils to the peritoneal cavity of mice.

**Conclusions:**

This study presents the potential utilization of a synthetic compound, as a lead for the development of novel anti-inflammatory drugs.

## Introduction

Cytokine regulation represents a potentially important therapeutic target in the inflammatory diseases. A variety of anti-cytokine strategies are being explored for the treatment of inflammation disorders. These include the neutralization of cytokines by soluble receptors or monoclonal antibodies and the activation of anti-inflammatory pathways by bioengineered [1]. However, the anti-cytokine drugs available to date are proteins, and suffer to a varying degree from the general disadvantages associated with protein drugs: limited stability, cellular penetration, cellular activity and oral absorption as well short half-life, rapid metabolism, immunogenicity and high costs of manufacturing. Therefore, small molecular anti-cytokine drugs, which target specific signaling and/or biosynthetic pathways of pro-inflammatory cytokines, would offer an attractive alternative to the treatment of inflammatory diseases [2].

A key element in this pathway is Mitogen-Activated Protein Kinase (MAPK) and nuclear factor kB (NF-κB). Activation of MAPK under a variety of stress stimuli like lipopolysaccharide (LPS) results in the phosphorylation and activation of other kinases and transcription factors such as NF-κB, which is responsible for the transcriptional regulation of genes that encode inflammatory cytokines. For example, the inhibition of these pathways blocks the production of cytokines such as TNF-α, at the transcriptional and translational levels [3]. Importantly, inhibition of the NF-κB pathway also culminates with the transcriptional inhibition of a novel and key cytokine, the High Mobility Group B1 (HMGB1) [4]. HMGB1 is a non-histone chromatin-associated protein that stabilizes DNA structure and modulates transcriptional activity. HMGB1 has been recognized as a potent pro-inflammatory cytokine actively secreted by innate immune cells in response to pathogenic products and released by injured or dying cells. Thus, HMGB1 occupies now a central role in the pathogenesis of both sterile and infectious inflammation [5]–[6].

An initial series of pyridyl-imidazole anti-inflammatory agents served as tools to elucidate the regulation of cytokine production in inflammation. The compound SB-203580, containing a pyridinyl imidazole group, is a practical example of small molecular anti-cytokine agents that inhibited the p38 MAPK and, consequently, decreased cytokine production [7]. Several others inhibitors unrelated to pyridinyl imidazole-based p38 inhibitors were discovered including triazanapthalenones, N,N′-diaryl ureas, N,N-diaryl ureas, benzophenones, pyrazole ketones, indole amides, diamides, quinazolinones, pyrimido[4,5-d]pyrimidinones and pyridylamino-quinazolines. It is of interest to note that a chemically diverse set of compounds inhibits p38 MAPK potently [8].

In a continuous effort to develop improved p38 MAPK inhibitors, Laufer et al synthesized compounds with isoxazole rings by bioisosteric replacement of the imidazole ring of SB-203580 aiming at diminishing its hepatotoxic effect. The compound 4-[3-(4-fluorophenyl)isoxazol-4-yl]pyridine displayed a promising anti-inflammatory activity, by suppressing cytokine release, lowering the affinity for cytochorme P450 and showing a decreased in the IC_50_ toward isolated p38 MAPK [9]. Another heterocycle with a potential to serve as an anti-inflammatory molecule is the 4,5-dihydroisoxazole, or isoxazoline. The compound VGX-1027 [(S,R)-3-phenyl-4,5-dihydro-5-isoxasole acetic acid] was the first of a new class of immune modulators accepted by the FDA (US Food and Drug Administration) that inhibits the production of pro-inflammatory cytokines by down-regulating the NF-κB and p38 MAPK pathways and up-regulating the ERK pathway [10].

The present work aimed at evaluating the anti-inflammatory potential of 4,5-dihydroisosaxoles, and thus we investigated the immunomodulatory effect of a synthetic 5-(4-pyridyl)-4,5-dihydroisosaxole derivative.

## Materials and Methods

### Ethics Statement

Animals were handled in strict accordance with good animal practice as defined by Animals Use Ethics Committee of UFRJ (Universidade Federal do Rio de Janeiro), with approval DFBCICB028. The study was conducted adhering to the institutiońs guidelines for animal husbandry.

### Synthesis of 3-(3-chloro-phenyl)-5-(4-pyridyl)-4,5-dihydroisoxazole

The 3-chloro-phenyl-aldoxime was synthesized from 20 mmol of the respective aldehyde and 60 mmol of NH_2_OH.HCl contained in 20 mL of solvent. The reaction medium was irradiated by microwave radiation for 30 min with potency of 160 W. In the following, the aldoxime reacted with trichloroisocyanuric acid (10 mmol) and triethylamine (20 mmol) for 24 h under stirring at room temperature to produce the corresponding hydroximoyl chlorides. Finally, 30 mmol of 4-vinylpiridine was added to the solution of dichloromethane and hydroximoyl chlorides. This reaction was under stirring for 24 h at 27°C. Soon after, the compound was separated by flash column chromatography, using as eluent hexane and ethyl acetate mixture in proportions of 3∶1, respectively. A yellow powder in 46% yield, mp 92°C was obtained. IR spectrum, cm^−1^: 1595 (νC=N of 4,5-dihydroisoxazole); 1555 (νC=C); 1078 (νC_sp2_–Cl). ^1^H NMR spectrum, δ, ppm, CDCl_3_: 3.28 (1H, dd, ^2^ *J*_H-H_ =16.6 Hz, ^3^ *J*_H-H_ =7.4 Hz); 3.84 (1H, dd, ^2^ *J*_H-H_ =16.6 Hz, ^3^ *J*_H-H_ =11.3 Hz); 5.76 (1H, dd, ^3^ *J*_H5-H4b_ =11.3 Hz, ^3^ *J*_H5-H4a_ =7.4 Hz); 7.35 (4H, m); 8.62 (2H, d, ^3^ *J*_H-H_ =6.0 Hz); 7.66 (1H, s); 7.56 (1H, d). ^13^C NMR spectrum, δ, ppm, CDCl_3_: 155.1; 42.8; 80.9; 149.8; 120.5; 150.3; 130.7; 130.5; 134.9; 126.9; 124,9; 130,2. Mass spectrum, [M^+^]: 258. Calcd for C_14_H_11_N_2_OCl: C, 65.0; H, 4.3; N, 10.8. Found: C, 64.7; H, 4.4; N, 10.8.

**Figure 1:**
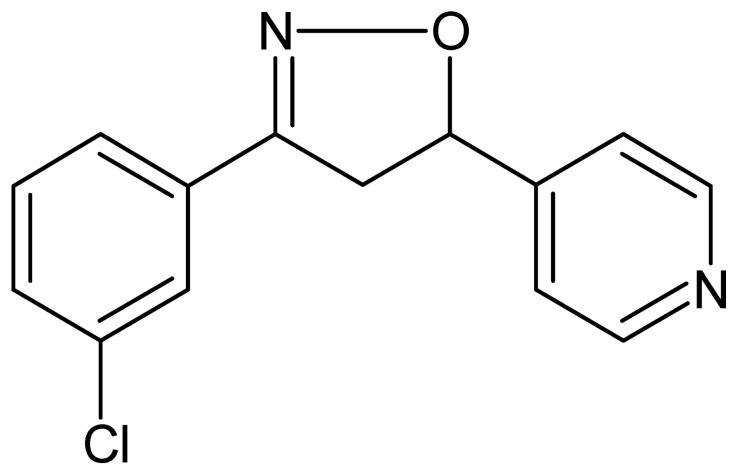
Chemical structure of 3-(3-chloro-phenyl)-5-(4-pyridyl)-4,5-dihydroisoxazole, DIC.

### Cell Culture and Viability

RAW 264.7 macrophage cell line (ATCC - TIB-71™) was grown at 37°C in RPMI 1640 (Sigma) medium supplemented with 10% Fetal Bovine Serum (FBS -Sigma) in a humidified atmosphere of 5% CO_2_. RAW 264.7 cells were plated at a density of 10^5^ cells/well in 96-well plates. The cells were treated with various concentrations of DIC for 24 h. Cell viability was determined by 3-(4,5-dimethylthiazol-2-yl)-2,5-diphenyl tetrazolium bromide (MTT colorimetric assay - Sigma) and the lactate dehydrogenase (LDH) leakage quantified by the CytoTox 96® kit (Promega) according to the manufacturer’s instructions.

### TNF-α, IL-6, and PGE_2_ Quantification

RAW 264.7 cells were pretreated with DIC at the indicated concentrations for 2 h and then stimulated with LPS (SIGMA − 100 ng/mL or 1 µg/mL) for 4 h to detected TNF-α levels or 24 h to IL-6 and PGE_2_. The levels of TNF-α, and IL-6 in the culture media were quantified using ELISA kits (Peprotech) according to manufacturer’s protocol. The levels of PGE_2_ in the culture supernatants were quantified using EIA kits (Cayman) according to manufacturer’s protocol.

**Figure 2:**
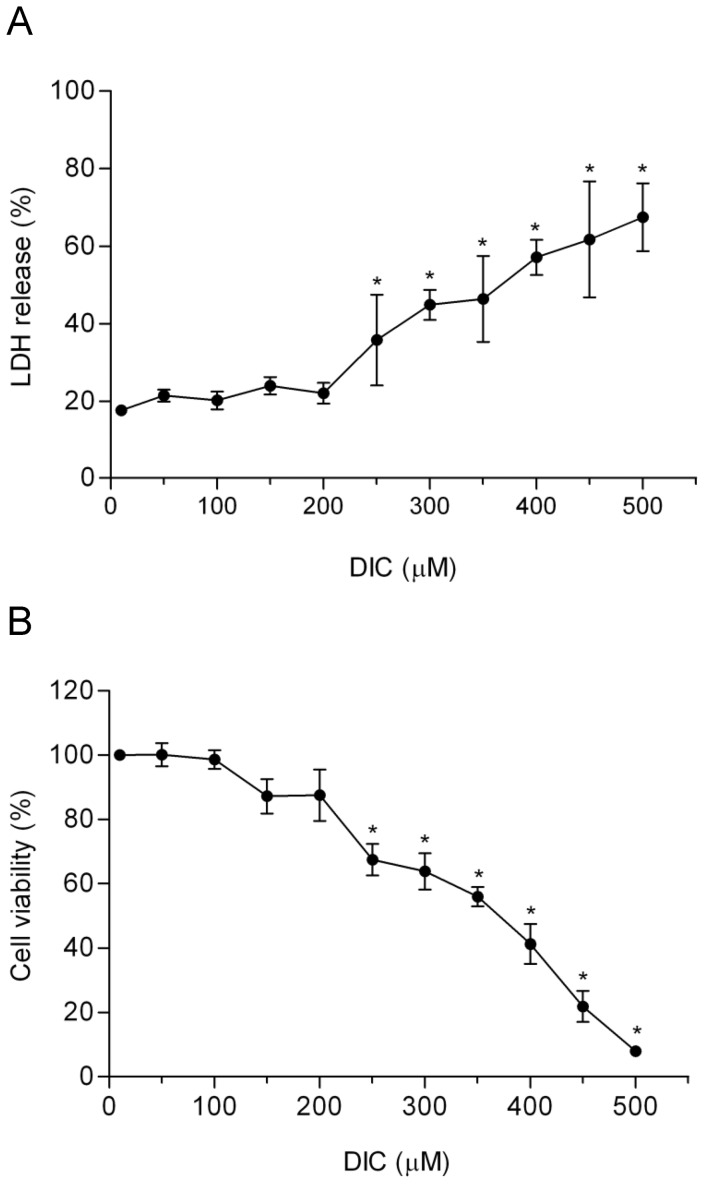
Effect of DIC on macrophage viability. RAW 264.7 macrophages were treated with DIC (from 10 µM to 500 µM) for 24 h. Cell viabilities were determined by LDH release (**A**) and MTT assay (**B**). Values represent means ± SD of three independent experiments. * Significant differences (p>0.05) between treated and untreated cells (250–500 µM), using unpaired t-test.

### Western Blot Analysis

RAW 264.7 cells (2×10^6^ cells/well in 6-well plates) were washed twice with cold PBS and harvested by scraping. The pellet was resuspended in extraction lysis buffer (200 mM HEPES/KOH, pH 7.9, 0.35 M NaCl, 20% (v/v) glycerol, 1% (v/v) Nonidet P-40, 1 mM MgCl_2_, 0.5 mM EDTA, 1 mM PMSF, 0.5 mM Na orthovanadate, 5 µg/mL aprotinin) and incubated on ice for 30 min. Cells were centrifuged at 14,000 rcf at 4°C for 10 min to pellet the cellular debris, followed by a quick freeze of the supernatants. Protein concentration was determined using the Bio-Rad protein assay reagent according to the manufacture’s instruction. Protein extract (30 µg) of treated or untreated cells was electroblotted onto a nitrocellulose membrane following separation on a 15% SDS–polyacrylamide gel electrophoresis. The immunoblot was incubated for 2 h with blocking solution (5% skim milk in TBS) at room temperature, followed by overnight incubation with various dilutions of the primary antibody (COX-2, p38, α-tubulin, phosphor-ERK and ERK, from Santa Cruz; phosphor-p38, phosphor-JNK, JNK and β-actin, from Cell Signaling). The blots were washed three times with Tween-20 0.05%/Tris-buffered saline (T-TBS) and incubated with peroxidase-conjugated secondary antibody for 1 h at room temperature. The blots were again washed three times with T-TBS, and the proteins visualized by enhanced chemiluminescence according to the instructions of the manufacturer (Amersham Life Science).

**Figure 3:**
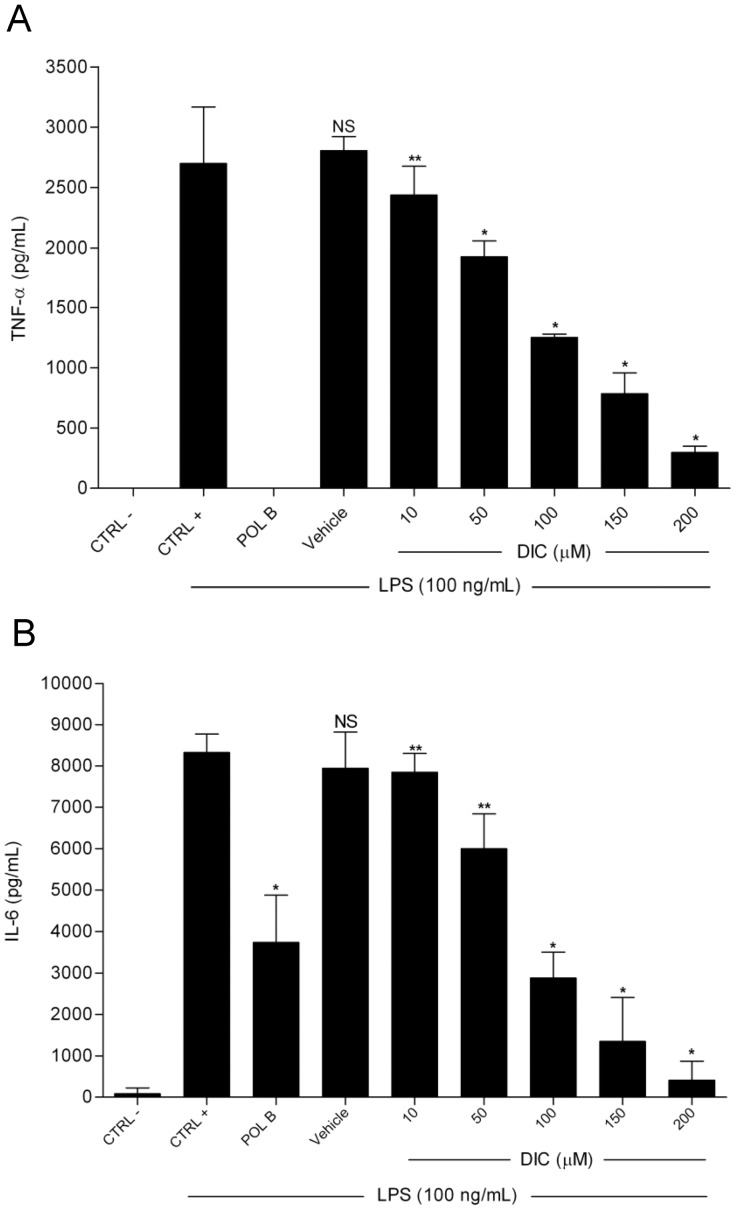
Effect of DIC on LPS-induced TNF-α and IL-6 production. **A** and **B**, following pretreatment with Polymyxin B (Pol B, 15 µg/mL), vehicle (DMSO 0.25%) or DIC (10−200 µM) for 2 h, the cells were treated with LPS (100 ng/mL) for 4 h (A) or 24 h (B). Negative control (CTRL −): cell medium only; Positive control (CTRL +): cells stimulated with LPS, only. TNF-α and IL-6 levels were assayed by ELISA. Values represent means ± SD of three independent experiments. NS, non-significant *vs* CTRL +; * p<0.05 *vs* vehicle; ** non-significant *vs* vehicle. Significances between treated groups were determined using unpaired t-test.

### Nuclear Extraction and Electrophoretic Mobility Shift Assay (EMSA)

RAW 264.7 cells were plated at a density of 2×10^6^ cells/well in 6-well plates, pretreated with DIC (150 and 200 µM) for 2 h, and then stimulated with LPS (1 µg/mL) for 1 h. The cells were washed once with PBS, scraped into 1 mL of cold PBS, and pelleted by centrifugation. Nuclear extracts were prepared as described previously by Holden [11]. Cell pellets were resuspended in buffer I (10 mM Tris–HCl pH 7.5, 0.15 M NaCl, 1.5 mM MgCl_2_, 0.65% NP-40, 0.5 mM PMSF, 10 mM DTT) mixed by vortex and left on ice for 15 min. Cells were centrifuged at 4°C, 12,000 rcf, 2 min and the resultant pellet lysed in buffer II (20 mM Hepes pH 7.9, 25% glycerol, 0.4 M NaCl, 1.5 mM MgCl_2_, 0.2 mM EDTA pH 8.0, 0.5 mM PMSF, 10 mM DTT), left on ice for 2 h, centrifuged at 4°C, 12,000 rcf, 10 min and the supernatant transferred to a new tube. Nuclear extracts (10 µg) were mixed with 200 c.p.m. of double-stranded ^32^P-end-labled-NF-κB oligonucleotide: 5′-AGTTGAGGGGACT-TTCCCAGGC-3′ (underline indicates the κB consensus sequence or a binding site for NF-κB/cRel homodimeric or heterodimeric complex). Binding reactions were carried out at 37°C for 30 min in 30 µL of binding buffer containing 10 mM Tris–HCl, pH 7.5, 100 mM NaCl, 1 mM EDTA, 4% glycerol, 1 µg of poly (dI-dC), and 1 mM DTT. DNA–protein complexes were separated from the unbound DNA probe on a native 6% polyacrylamide gels, run at 200 V in 0.5×Tris Boric Acid EDTA (TBE) buffer. Gels were vacuum-dried for 1 h at 80 °C and exposed to X-ray film at −70°C overnight.

**Figure 4:**
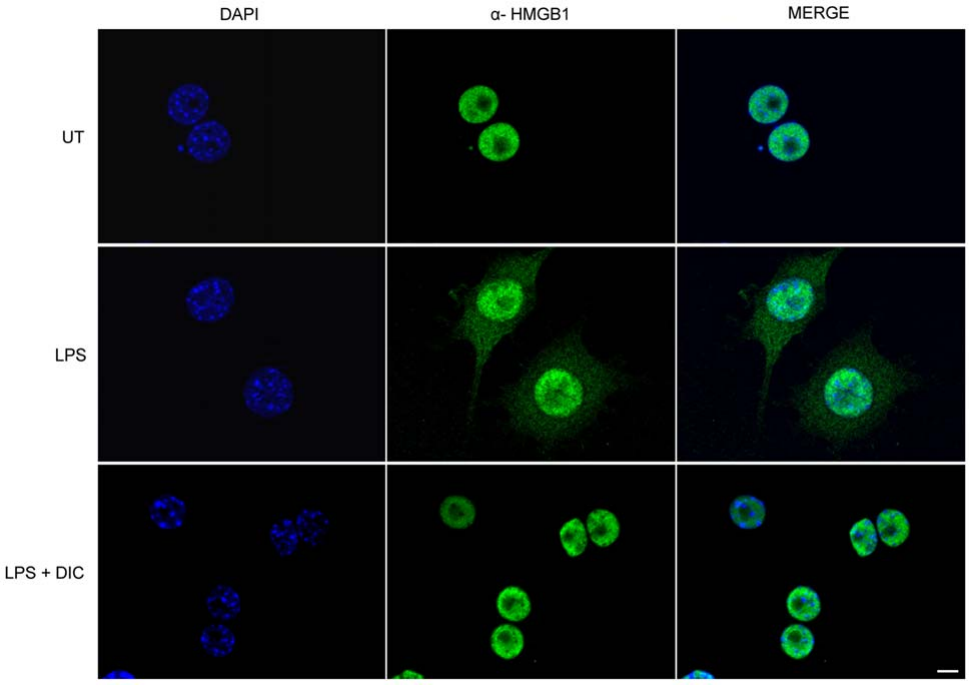
Effect of DIC on nuclear translocation of HMGB1. RAW 264.7 macrophages were pretreated with DIC 200 µM for 2 h prior to addition of LPS (1 µg/mL) for 24 h. Intracellular HMGB1 was visualized with green immunofluorescent FITC-staining. Untreated cells (UT); LPS-stimulated cells (LPS); DIC-treated cells stimulated with LPS (LPS + DIC).

### Immunocytochemistry

The immunocytochemistry approach was used to investigate the immunolocalization of HMGB1 and p65. RAW 264.7 cells were plated on glass coverslips in 24-well plates (50,000 cells/well) and cultured in RPMI 1640 medium supplemented with 10% FBS, under standard culture conditions of 5% CO_2_. The cells were pretreated with 200 µM of DIC or vehicle (DMSO 0.25%) for 2 h and then stimulated with LPS (1 µg/mL) for 24 h, for HMGB1 analysis or 1 h, for p65 analysis. For immunofluorescence detection of proteins, cells were fixed in 4% paraformaldehyde (Sigma) for 10 min and then, blocked for 1 h in 5% Bovine Serum Albumin (BSA – Sigma) diluted in Tween-20 0.5%/Phosphate-buffered saline (T-PBS) followed by permeabilization in PBS containing 10% of Triton X-100 for 30 min. Glass coverslips were incubated with monoclonal primary antibodies (HMGB1, Abcam; p65, Cell Signaling) overnight at 4 °C. After washing, an Alexa Fluor 488 (FITC-staining) conjugated anti-rabbit antibody (Invitrogen) was added for 1 h at room temperature. An irrelevant anti-IgG1 and secondary antibodies only were used as negative controls (data not shown). The chamber slides were mounted in Prolong Gold antifade reagent with DAPI (Invitrogen). Fluorescence images were obtained with a Zeiss Axio Observer.Z1 invert microscope equipped with 1006× objective lens and an AxioCam MRm camera, in the ApoTome mode.

### Thioglycollate-Induced Peritonitis in Mice

These experiments were performed according to the method described by Savill [12]. Female Balb/c, 8 weeks old (CECAL, FIOCRUZ, Rio de Janeiro) were treated intraperitoneally (i.p.) with DIC (5 mg/kg) or vehicle (DMSO 2.4%), 30 min before the administration of 1 mL of 3% thioglycollate (Difco-BD Biosciences). After 4 h, animals were killed by cervical dislocation and the peritoneal cavity was washed with 3 mL of cold PBS. The peritoneal exudates were retrieved and their volume was measured. Total cell migration was counted using a Neubauer chamber. Exudates (100 µL) were mounted on glass slides by cytospinning for 10 min at 1,000 rcf (Cytospin 3, Shandon Scientific) and stained with hematoxilin-eosin for differential cell count. Two hundred cells per sample were counted.

**Figure 5:**
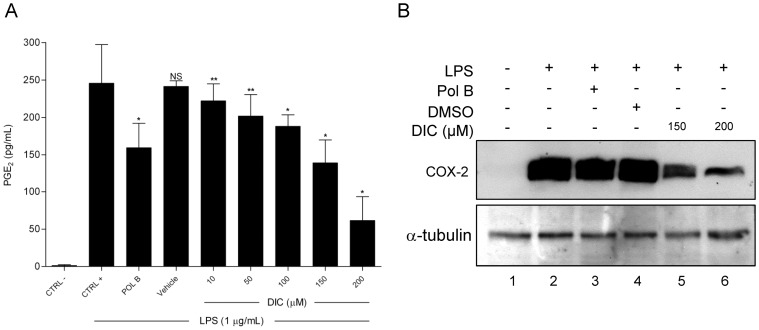
Effect of DIC on LPS-induced PGE_2_ production. (**A**) RAW 264.7 macrophages were pretreated with DIC 200 µM for 2 h prior to addition of LPS (1 µg/mL) for 24 h and then PGE_2_ levels were determined by EIA. The values shown are means ± SD of three independent experiments. NS, non-significant *vs* CTRL+; * p<0.05 *vs* vehicle; ** non-significant *vs* vehicle. Significances between treated groups were determined using unpaired t-test. (**B**) Protein levels of COX-2 were determined by western blot analysis of cellular protein extract (upper panel). A representative immunoblot out of three independent experiments were shown.

### Statistical Analysis

Results were expressed as the mean ± S.D. of triplicate experiments. Statistically significant values were compared using t-test, and *p* values of less than 0.05 were considered statistically significant. GraphPad Prism 5.0 statistical software (NIH software) was used.

**Figure 6:**
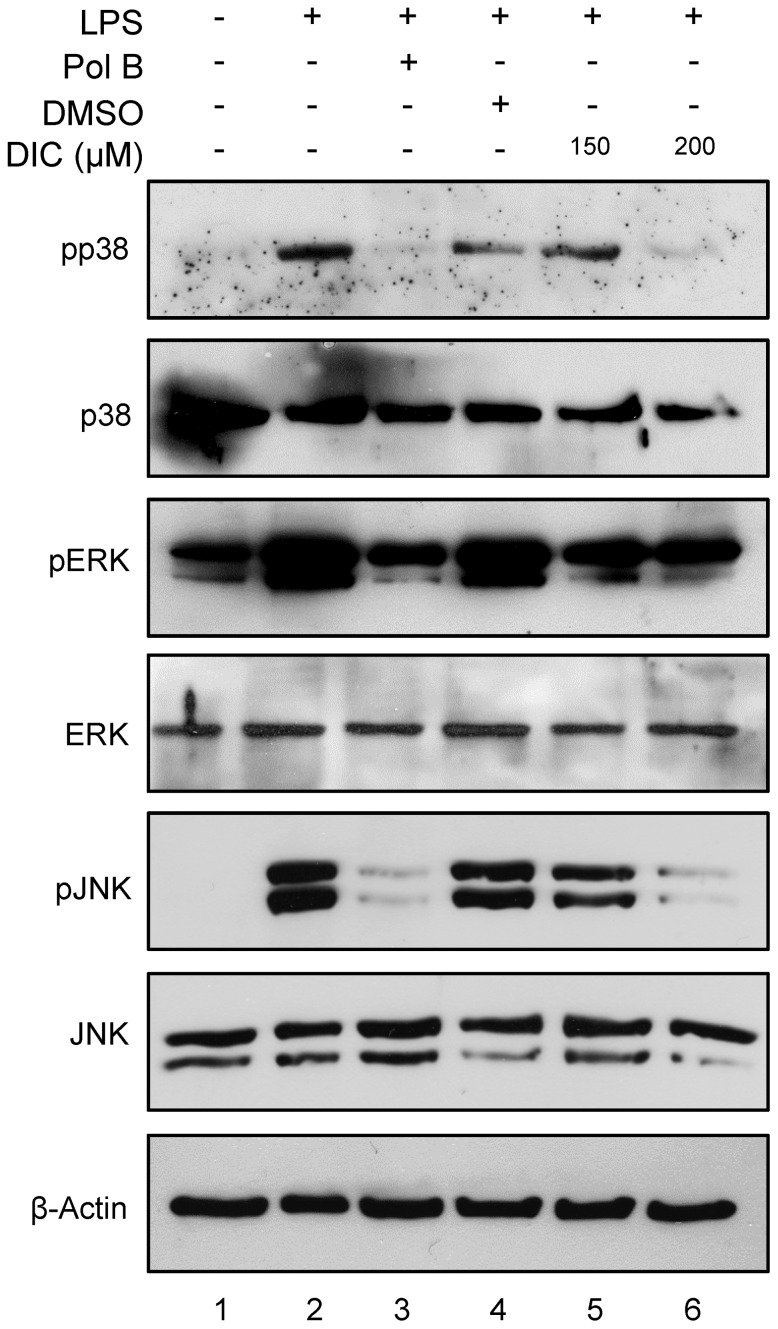
Effect of DIC on the MAPK pathway. RAW 264.7 macrophages were pretreated with 200 µM of DIC for 2 h prior to addition of LPS (1 µg/mL) for 15 min, and then the whole cell lysate was analyzed by western blot using antibodies against the phosphorylated (activated) and unphosphorylated MAPK. The data shown are representative of three independent experiments.

## Results

### Synthesis of 3-(3-chloro-phenyl)-5-(4-pyridyl)-4,5-dihydroisoxazole

The compound 3-(3-chloro-phenyl)-5-(4-pyridyl)-4,5-dihydroisoxazole**,** or DIC (Figure 1) was synthesized from a cycloaddition reaction of 3-chloro-benzonitrile oxide to 4-vinylpiridine [13]. As a result of this reaction, a yellow powder (46% yield) was obtained. DIC, which is a five-membered heterocyclic compound containing a N-O bond was characterized by FTIR, mass spectroscopy and ^1^H-NMR and ^13^C-NMR, as described in the materials and methods section.

**Figure 7:**
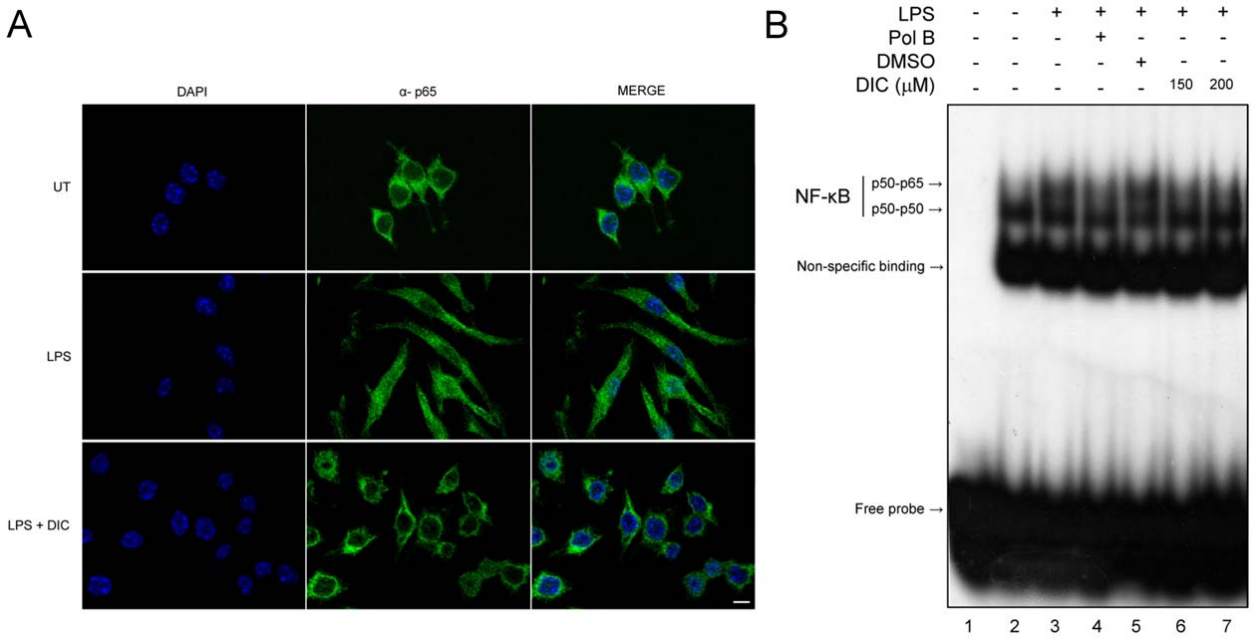
Effect of DIC on the activation of NF-κB signaling pathway. (**A**) RAW 264.7 macrophages were grown on coverslips, pretreated with DIC at a concentration of 200 µM for 2 h and stimulated with LPS (1 µg/mL) for 1 h. FITC-immunostained for NF-κB/p65 viewed under a fluorescence microscope in the ApoTome mode. Untreated cells (NT); LPS-stimulated cells (LPS); DIC-treated cells stimulated with LPS (LPS + DIC). Scale bar: 10 µm. (**B**) EMSA analysis: nuclear extracts were prepared from unstimulated cells (lane 2); cells stimulated with LPS (1 µg/mL - lane 3); cells pretreated with Polymyxin B (15 µg/mL - lane 4), cells treated with vehicle only (DMSO 0.25% - lane 5); cells treated with increasing concentrations of DIC (lanes 6 and 7) for 2 h and stimulated with LPS for 1 h. The analysis was based on the DNA binding by the active NF-ΚB heterodimer p50–p65. DNA binding inhibition of the p50–p65 heterodimers by DIC is observed (compare lanes 3 and 5 with lanes 6 and 7; the intensities of the upper bands [p50–p65 arrow] in lanes 6 and 7 are significantly lower).

### Effect of DIC on Macrophage Toxicity

Two different cytotoxicity tests (LDH and MTT) were used to evaluate the biocompatibility of DIC. The LDH test measures only severe cell damage and enzyme release upon damage, whereas the MTT test measures the mitochondrial activity of the cells [14].

*In vitro* cytotoxicity was determined in RAW 264.7 macrophages treated with DIC for 24 h at concentrations ranging from 10 to 500 µM. Concentrations of DIC up to 200 µM did not display any cellular toxicity against the cells (measured by both methods, LDH or MTT). Alternatively, higher concentrations of DIC (from 250 µM to 500 µM) were toxic to the cells (Figure 2 A and B).

**Figure 8:**
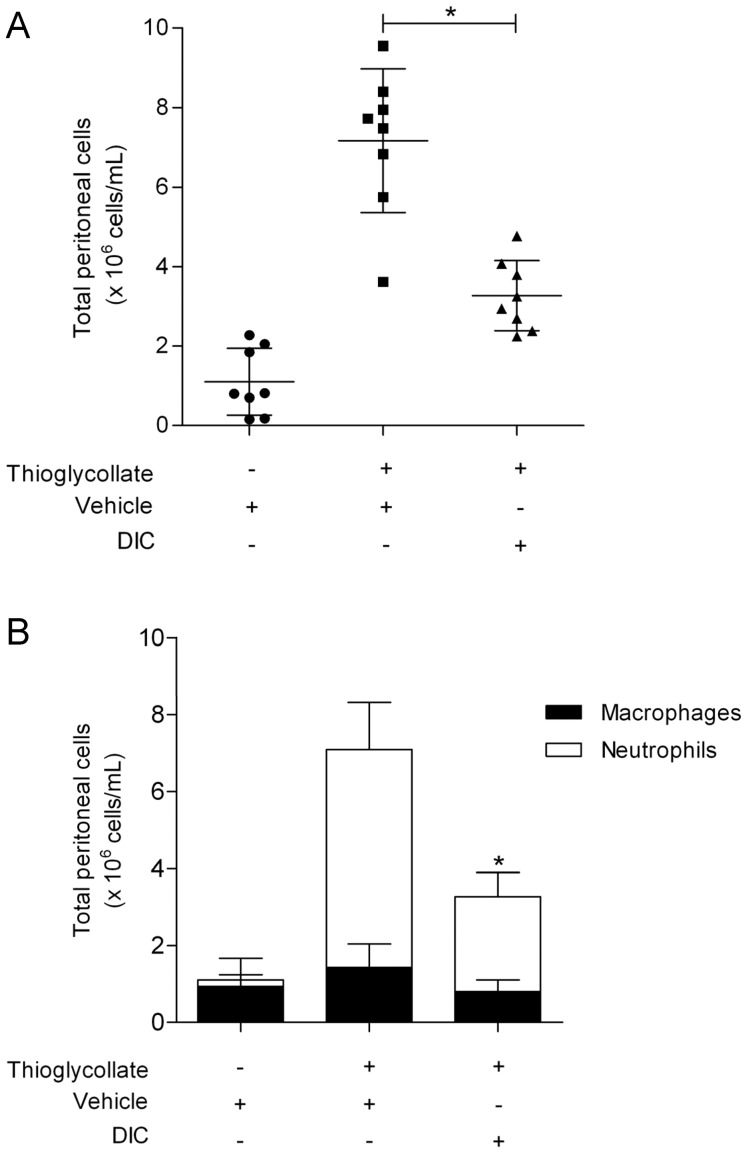
Effect of DIC on cell migration in thioglycollate-induced peritonitis in mice. (**A**) DIC (5 mg/kg) or vehicle (DMSO 2.4%) was administered intraperitoneally 30 min before the thioglycollate administration. Mice were sacrificed after 4 h of thioglycollate-induced peritonitis. Total cell migration was counted using a Neubauer chamber. (**B**) Differential cell count was evaluated by Cytospin. Data represent mean ± S.D. from at least 8 animals per group. * P<0.05 (significances between treated groups were determined using unparied t-test).

### Effect of DIC on LPS-induced TNF-α and IL-6 Production

To determine the effect of DIC on the production of pro-inflammatory cytokines following LPS treatment, ELISAs were performed using cell culture supernatants. During incubation, cells in the resting state produced undetectable levels of TNF-α, but 81.5 pg/mL of IL-6. When the cells were exposed to LPS, TNF-α production increased about 3.000 fold (2.700 pg/mL) and IL-6 increased about 100 fold (8.300 pg/mL) over the basal level (Figure 3 A and B). It is important to note that DIC inhibited the production of both cytokines from concentrations ranging from 50 to 200 µM (Figure 3 A and B). However, DIC at 200 µM concentration was able to almost completely abolish the production of TNF-α and IL-6 to 89.4% and 94.9%, respectively (Figure 3 A and B). DMSO at 0.25% and Polymyxin B (15 µg/mL) were used as controls.

### Inhibition of LPS-induced Nuclear Translocation of HMGB1 by DIC

Besides its canonical DNA transactions within the nucleus, HMGB1 was recently recognized as an inflammatory mediator actively secreted as a cytokine by macrophages and other inflammatory cells upon cell injury and infection [5], [15].

Since we showed that DIC inhibited the release of classical cytokines such as TNF-α and IL-6 (Figure 3 A and B), we decided to investigate whether DIC could also interferer in the secretion of HMGB1 by LPS-activated macrophages. Immunofluorescence microscopy showed that HMGB1 remained in the nuclei of macrophages when the cells received no inflammatory (LPS) stimulus (Figure 4, upper panels, or UT). However, HMGB1 was readily translocated from the nucleus to the cytoplasm of macrophages that were stimulated with LPS (Figure 4, central panels, or LPS). Importantly, when macrophages were stimulated by LPS and treated with DIC, we clearly observed the retention of HMGB1 in the nuclei of the cells (Figure 4, bottom panels, or LPS + DIC).

### Effect of DIC on LPS-induced PGE_2_ Production

To investigate the effect of DIC on LPS-induced PGE_2_ production by RAW 264.7 macrophages, cell culture media were harvested, and the levels of PGE_2_ were measured after exposure of LPS for 24 h. The treatment with DIC (10 to 200 µM) significantly inhibited LPS-induced PGE_2_ production, corresponding to 75.6% at 200 µM (Figure 5A).

Since DIC was found to inhibit PGE_2_ production, we decided to investigate whether this inhibitory effect was related to the regulation of COX-2 expression. Western blot analysis showed that COX-2 protein levels were markedly up-regulated in response to LPS for 24 h (Figure 5B, upper panel, lane 2). Importantly, DIC significantly inhibited the expression of COX-2 (lanes 5 and 6). DIC has no effect on the expression of α-tubulin (Figure 5B, bottom panel).

In addition, LPS leads to COX-2 activation, which in turn leads to the production of PGE_2_ (Figure 5A, CTRL+); we see that in the presence of Pol B, only 10% (measured by densitometry) of COX-2 was inhibited (Figure 5B, lane 3); thus, we believe that the 35% reduction in PGE_2_ release by Pol B is reflected by the slight reduction in COX-2 expression.

### Inhibition of LPS-induced MAPK Activation by DIC

MAPKs play critical roles in the regulations of cell growth and differentiation and in the cellular responses to cytokines and other stresses. In addition, MAPKs are also known to be important for the transcriptional activation of NF-κB [16]. To investigate whether the inhibition of the inflammatory response by DIC was mediated by the MAPK pathway, we examined the effect of DIC on the LPS-stimulated phosphorylation of p38 MAPK, ERK1/2 and JNK in RAW 264.7 cells. Western blot analysis showed that DIC was able to inhibit p38, ERK and JNK phosphorylation (Figure 6, compare: lanes 2 and 6 for pp38; lanes 2, 5 and 6 for pERK; lanes 2, 5 and 6 for pJNK). Constitutive levels of p38, ERK and JNK are shown (p38, ERK and JNK panels). The expression levels of actin remained unaltered after the treatments (Figure 6, bottom panel).

### Effect of DIC on the Activation of NF-κB Signaling Pathway

By employing immunofluorescence microscopy, we found that p65 was localized to the cytoplasm of macrophages before LPS stimulation (Figure 7A, upper panels, or UT). However, 1 h after stimulation with LPS at 1 µg/mL, the majority of intracellular p65 translocated from the cytoplasm to the nucleus, demonstrated by strong p65 staining within the nucleus (Figure 7A, central panels, or LPS). When macrophages were stimulated with LPS and treated with 200 µM of DIC, LPS-induced nuclear translocation of p65 was strongly inhibited, as shown by the lack of p65 staining within the nuclei of the cells (Figure 7A, bottom panels, or LPS + DIC). To further examine the effect of DIC on LPS-induced NF-κB translocation, we performed EMSA using an oligonucleotide probe containing a NF-κB response element. We showed that treatment of macrophages with LPS alone led to an increase in NF-κB-(p50–p65)-DNA binding activity (Figure 7B, lane 3). As expected, pretreatment with Polymyxin B (a scavenger of LPS) was able to attenuate NF-κB binding (Figure 6B, lane 4), and DMSO had no effect in DNA binding (Figure 7B, lane 5). However, pretreatment with DIC prior LPS stimulation significantly inhibited NF-κB-(p50–p65)-DNA binding activity (Figure 7B, lanes 6 and 7).

### Effect of DIC on Cell Migration in Thioglycollate-induced Peritonitis in Mice

To explore the anti-inflammatory effect of DIC *in vivo* we first studied cell recruitment in the peritoneal cavity of mice. The animals were pretreated with DIC (5 mg/kg) or vehicle (DMSO 2.4%) i.p. and 30 min later the thioglycollate was injected i.p. to induce peritonitis. Similar to previous data [17], 4 h after thioglycollate injection, we found an increase of inflammatory cells (Figure 8A), mainly consisting of neutrophils (81%), as shown in Figure 8B. In the control group without thioglycollate injection, only resident macrophages were found in the peritoneal cavity (Figure 8B, first column). Pretreatment with DIC followed by thioglycollate injection decreased the recruitment of neutrophils (56.5% of inhibition when compared with the thioglycollate group) as shown in Figure 8 A and B, third column.

## Discussion

Pro-inflammatory mediators such as cytokines are strictly related to immuno-pathogenic processes of a variety of inflammatory diseases. These evidences triggered the development of a new strategy for the treatment of immune-inflammatory disorders, based on the neutralization of the action of pathogenic cytokines. Such neutralization may act through the use of monoclonal antibodies or soluble receptors. However, this type of therapy still shows disadvantages due to the fact that the administration is based on protein molecules [2].

In this regard, there is a growing need for the development of synthetic small molecular anti-cytokine agents. Today, the MAPK and NF-κB pathways are important targets for these drugs, since they have seen to be over activated in a variety of inflammatory diseases, culminating in the over expression of cytokines [3].

With the goal of generating small molecular anti-cytokine agents that could mediate inflammatory responses, in this work we characterized the anti-inflammatory activity of a synthetic 3-(3-chloro-phenyl)-5-(4-pyridyl)-4,5-dihydroisoxazole, or DIC, a five-membered heterocyclic compound containing a N-O bond. The 4,5-dihydroisoxazoles are easily synthesized through a cycloaddition reaction [13], [18]–[19]. This reaction produce different 4,5-dihydroisoxazoles disubstituted which can present different bioactivities as antifungal, anticancer, antibacterial, antiparasitic and anti-inflammatory activity [10], [20]–[28]. Although the anti-inflammatory role of the 4,5-dihydroisoxazoles is still not completely understood, it is suggested that the heterocyclic component plays a major role in favoring the ideal location of its substituent in space, in a way that it facilitates and allows chemical interactions between the drug and its biological targets [9], [23]. 4,5-dihydroisoxazol derivatives can present anti-inflammatory activities due to the nature of the substituent [7], [22]–[23]. The literature has reported that derivatives of this heterocyclic compound such as the VGX-1027 [7] present an immunomodulatory activity that inhibits the MAPK/NF-κB signaling pathways. Additionally, this type of activity has been reported for several derivatives containing pyridines as substituent, although the number of papers investigating these derivatives is still limited (see patent at www.wipo.int/patentscope/search/en/wo2005034952).

The present paper reports the characterization of the anti-inflammatory activity of a new derivative of 4,5-dihydroisoxazol containing a phenyl and pyridyl radicals as substituent at positions C3 and C5 of the heterocyclic, respectively.

In the context of the innate immunity, it is known that components of the Gram-negative bacteria wall such as LPS, stimulate macrophages to produce cytokines like TNF-α, IL-1β, IL-6, IL-12, HMGB1, several chemokines, and also induce the synthesis of enzymes as iNOS, COX-2 and others [29]–[30]. These pro-inflammatory mediators and enzymes play key roles in the pathogenesis of several chronic and acute inflammatory diseases. Consequently, the use of synthetic drugs to modulate the production of these mediators becomes an alternative therapeutic strategy [2]. In this regard, we showed in this paper that DIC possesses anti-inflammatory properties by inhibiting the production of TNF-α, IL-6, and PGE_2_ and in macrophages stimulated with LPS. Moreover, our data also suggested that DIC acts as an anti-inflammatory compound through the inhibition of HMGB1 translocation and subsequent secretion by macrophages.

It is well established that NF-κB is the main transcriptional regulator of a variety of genes involved with immune responses [31]. Another key element to the regulation of cytokine production is the MAPKs, which are strictly related with the activation of NF-κB, through the action of MSK1 [16]. Inhibitors of p38 MAPK and NF-κB block the production of IL-1, TNF-α and HMGB1 at the transcriptional and translational levels, being effective in the treatment of chronic inflammatory diseases [3]–[4]. Our data demonstrated that LPS-activated macrophages that were treated with DIC resulted in inhibition of the MAPK pathway, with the subsequent inhibition of the NF-κB pathway. Thus, we showed that treatment of macrophages with DIC inhibited the translocation of p65 from the cytoplasm to the nucleus. In this context, the absence of p65 in the nucleus ultimately compromised the DNA binding function of NF-κB, as confirmed by our EMSA assays. These set of data led us to believe that DIC is somewhat affecting the MAPK/NF-κB signaling pathways.

In this study we described that DIC is not cytotoxic and possesses significant anti-inflammatory activity. It is also important to bear in mind that the obtainment of the synthetic DIC is fast and of low cost. This summarizes the potential value of the 5-(4-pyridyl)-4,5-dihydroisosaxole as a lead compound for the development of a novel anti-inflammatory drug.
